# Cerebrotendinous xanthomatosis: A complex interplay between a clinically and genetically heterogeneous condition

**DOI:** 10.1111/ene.70006

**Published:** 2025-01-06

**Authors:** Emily O'Keefe, Matthew Kiernan, William Huynh

**Affiliations:** ^1^ Department of Neurology Gosford Hospital Gosford New South Wales Australia; ^2^ Brain and Mind Centre, University of Sydney Camperdown New South Wales Australia; ^3^ Department of Neurology Royal Prince Alfred Hospital Sydney Australia; ^4^ Neuroscience Research Australia (NeuRA) Sydney New South Wales Australia; ^5^ Department of Neurology Prince of Wales Hospital Randwick New South Wales Australia

**Keywords:** cerebrotendinous xanthomatosis, spastic paraparesis, transcranial magnetic stimulation, variant of uncertain significance

## Abstract

**Background and Purpose:**

Cerebrotendinous xanthomatosis (CTX) is a rare autosomal recessive lipid storage disease characterized by abnormal bile acid synthesis. It often presents with systemic and neurological manifestations; however, atypical presentations can lead to significant diagnostic challenges. This case report highlights the diagnostic complexities and management considerations in a patient with an uncommon presentation of CTX.

**Methods:**

We present a patient with a 25‐year history of spastic paraparesis, initially suggestive of hereditary spastic paraplegia (HSP), ultimately diagnosed with CTX associated with a novel CYP27A1 variant of uncertain significance (VUS).

**Results:**

A 53‐year‐old Greek woman presented with a 25‐year history of slowly progressive spastic paraparesis. Initial investigations were largely unremarkable, leading to a presumptive diagnosis of a hereditary spastic paraplegia (HSP)‐like syndrome. After 5 years of slow disease progression, the patient developed right ankle swelling. MRI revealed significant enlargement of the Achilles tendon, suggestive of xanthoma infiltration. Subsequent genetic testing identified a homozygous variant of uncertain significance (VUS) in the CYP27A1 gene. Biochemical analyses revealed elevated cholestanol levels and cholestanepentol glucuronide in urine, confirming the diagnosis of CTX. Treatment with chenodeoxycholic acid stabilized her condition over 3 years, but advanced disease limited efficacy in improving disability.

**Conclusion:**

This case highlights the diagnostic challenges associated with CTX, stemming from its relative rarity and significant clinical heterogeneity. It emphasizes the importance of a comprehensive approach combining clinical suspicion, imaging, genetic testing and biochemical analyses for accurate diagnosis, interpretation of VUS and management of rare conditions like CTX.

## CASE DESCRIPTION

A 53‐year‐old Greek woman presented with a 25‐year history of slowly progressive spastic paraparesis. Symptom onset was asymmetrical at age 30, affecting the right lower limb before involving the left within months, leading to recurrent falls. She denied upper limb, bulbar or respiratory symptoms, reporting only mild bladder overactivity. Her medical history included well‐controlled hypothyroidism, with no family history of neurological disorders.

Initial examination at age 48 revealed prominent lower limb spasticity, hyperreflexia and upgoing plantar responses, although no clonus was elicited. Mild weakness was noted in hip abduction and flexion, and knee flexion, with moderate‐to‐severe symmetric weakness in ankle dorsiflexion. Upper limb examination demonstrated brisk reflexes, normal tone without spasticity and preserved strength. Sensory examination, including light touch, pinprick, vibration and proprioception, was unremarkable. Cranial nerve examination was normal.

Neuroimaging investigations were largely unremarkable, with brain MRI revealing two small lateral convexity meningiomas and spine MRI showing no structural abnormalities. Initial targeted massively parallel sequencing of neuromuscular genes in 2014 was unremarkable. Cerebrospinal fluid analyses, nerve conduction studies and needle electromyography were normal, with no evidence of lower motor neuron pathology.

Transcranial magnetic stimulation revealed bilaterally inexcitable motor cortices, indicating impaired corticospinal tract function and upper motor neuron excitability with absent motor evoked potentials [1] Coupled with the clinical examination and preserved lower motor neuron function on electromyography, these findings supported a provisional diagnosis of a pure upper motor neuron syndrome, such as hereditary spastic paraplegia or primary lateral sclerosis.

**FIGURE 1:**
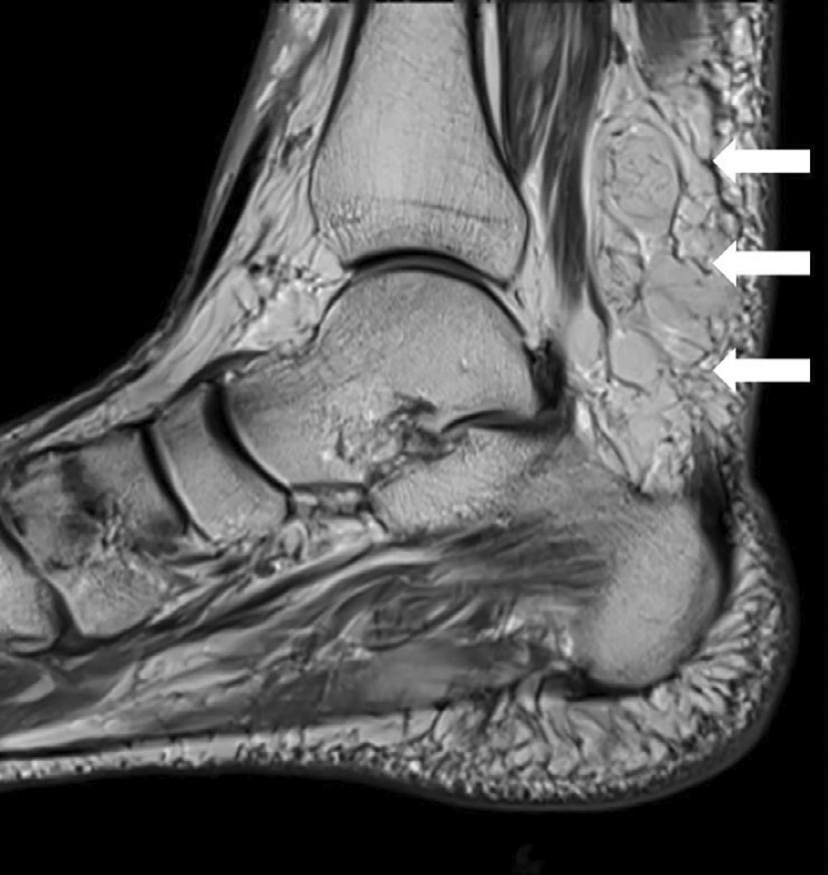
Sagittal plane of T1‐weighted MRI showed significant enlargement of the Achilles tendon (diameter ~ 2.8 cm) extending from the calcaneus into the musculotendinous junction, suggestive of xanthoma infiltration.

Management over the ensuing years was primarily symptomatic, including analgesia, neuropathic medications, baclofen and physical therapy. After 5 years of slow, lower limb‐predominant progression, the patient became wheelchair‐dependent. At a routine clinical review, she reported new subacute right ankle swelling and pain. Examination revealed a prominent subcutaneous soft tissue swelling overlying the right Achilles tendon. MRI of the right ankle demonstrated significant Achilles tendon enlargement, suggestive of xanthoma infiltration (Figure 1).

Further neuromuscular gene panel testing in 2019 identified a homozygous CYP27A1 missense variant (c.991G>C, p.(Glu331Gln), exon 5) reported to be of uncertain significance (VUS). This expanded panel included CYP27A1 gene, which was absent in the original 2014 neuromuscular sub‐exomic sequencing. In silico analysis with VarSome suggested that the variant was disease causing. Subsequent biochemical analyses revealed elevated serum cholestanol (24.50 μmol/L; normal range: 0.00–13.80 μmol/L) and urinary cholestanepentol glucuronide levels.

Applying the American College of Medical Genetics and Genomics (ACMG) guidelines, evidence supporting pathogenicity included the variant's absence from population databases like gnomAD, location in a functional domain with nearby pathogenic variants p.(Val337Ala) and p.(Thr339Met), deleterious effect predictions by in silico tools, elevated serum cholestanol and urinary bile alcohols, and the patient's highly specific CTX phenotype. [2] Based on these biochemical, genetic and clinical findings, and the presence of one strong, two moderate and two supporting ACMG criteria, the novel CYP27A1 variant was classified as likely pathogenic, resulting in the diagnosis of CTX.

Treatment with chenodeoxycholic acid (CDCA) 750 mg daily and Ox Bile acid 500 mg daily was initiated, alongside ongoing symptomatic management. Over the following 3 years, the patient remained clinically stable with no significant neurological or disability progression. CDCA treatment improved her biochemical profile, reducing serum cholestanol levels and normalizing urinary bile alcohol excretion.

## DISCUSSION

This case highlights the diagnostic challenges associated with cerebrotendinous xanthomatosis (CTX), stemming from its relative rarity and significant clinical heterogeneity. [3] The patient's 25‐year course illustrates the propensity of CTX to mimic other neurodegenerative disorders like HSP and PLS. [4] The absence of classic features at presentation, including cataracts, tendon xanthomas, chronic diarrhoea and ataxia, often delays diagnosis and commencement of disease‐modifying agents. [5] The initial negative gene panel testing, which did not include CYP27A1, further contributed to diagnostic delay in this case. This emphasizes the importance of a comprehensive differential diagnoses and stepwise exclusion of treatable metabolic conditions like CTX with biomarker testing in the absence of targeted gene panels or exome sequencing.

This case also illustrates the challenges in interpreting VUS, which are common in rare diseases like CTX, where limited genotype–phenotype correlations can lead to ambiguity in predicting pathogenicity. [4, 5] The laboratory classification of the CYP27A1 variant as a VUS, in the absence of further supporting evidence, highlights this complexity. Resolving VUS relies on reconciling clinical, imaging and laboratory findings, and applying standardized variant classification criteria like the ACMG guidelines. [6] The emergence of xanthomatosis proved pivotal in prompting re‐evaluation of the diagnosis and the classification of the CYP27A1 VUS as pathogenic, when combined with disease‐specific biochemical findings, including substantially elevated serum and urine cholestanol. [3, 5, 7]

The patient's minimal response to treatment aligns with evidence showing poorer outcomes in advanced CTX despite adequate biochemical control. [3, 6] While arresting further progression remains beneficial, advanced disease stage predicts limited treatment efficacy. [3, 6] Therefore, ongoing supportive care is essential to optimize quality of life given the irreversible neurological damage.

Quantifying treatment benefits in advanced disease stages remains challenging. However, research focusing on prognostic models and biomarkers may clarify complex genotype–phenotype relationships to predict outcomes and tailor care. Developing validated tools for early detection and timely treatment initiation is crucial, as intervening before irreversible neurological injury accrues is key to mitigating morbidity. [6] Large‐scale collaborative databases paired with functional assays and family member testing could assist in categorizing variants along the spectrum from benign to pathogenic and initiating management. Implementing population‐based screening initiatives in high‐risk groups, such as children with juvenile cararacts, may also promote earlier detection and treatment. [5] Such comprehensive processes are crucial for mitigating uncertainty, ensuring accurate diagnosis and management, and ultimately improving outcomes for CTX patients.

Overall, this case provides valuable insights into the diagnostic, genetic, therapeutic and clinical nuances of recognizing and managing rare conditions like CTX. A meticulous approach that combines clinical suspicion with appropriate imaging, genetic testing and biochemical analyses may prove invaluable in overcoming diagnostic pitfalls. This enables the timely initiation of potential disease‐modifying therapies that remain essential for optimal functional outcomes.

## AUTHOR CONTRIBUTIONS

**Emily O'Keefe:** Writing – review and editing; writing – original draft. **Matthew Kiernan:** Supervision; writing – review and editing. **William Huynh:** Supervision; conceptualization; writing – review and editing.

## CONFLICT OF INTEREST STATEMENT

The authors have no relevant financial or non‐financial interests to disclose.

## ETHICS APPROVAL

The patient provided informed consent for the publication of this case report.

## Data Availability

The data that support the findings of this study are available on request from the corresponding author. The data are not publicly available due to privacy or ethical restrictions.
